# Factors associated with COVID-19 preventive behaviors among taxi drivers in Bangkok

**DOI:** 10.3389/fpubh.2023.1049877

**Published:** 2023-01-27

**Authors:** Amornrat Deesua, Wonpen Kaewpan, Surintorn Kalampakorn, Jutatip Sillabutra

**Affiliations:** ^1^Department of Public Health Nursing, Faculty of Public Health, Mahidol University, Bangkok, Thailand; ^2^Department of Biostatistics, Faculty of Public Health, Mahidol University, Bangkok, Thailand

**Keywords:** COVID-19, health behavior, taxi driver, occupational health, public transportation

## Abstract

**Objectives:**

This paper aimed to identify factors associated with COVID-19 preventive behaviors among taxi drivers in Bangkok.

**Methods:**

This cross-sectional study included 401 taxi drivers. Data were analyzed using descriptive statistics. The association between predisposing factors, enabling factors, and reinforcing factors with COVID-19 preventive behaviors was analyzed by using analysis of variance and Pearson's Product Moment Correlation. Multiple linear regression analysis was used to determine the influencing factors in predicting COVID-19 preventive behaviors of taxi drivers.

**Results:**

The present findings revealed that income adequacy, support from family, co-workers, and healthcare professionals, perceived susceptibility, severity, benefits, barriers, and health motivation, accessibility to personal protective equipment for COVID-19 and preventative measures against COVID-19 from other agencies were associated with good COVID-19 preventive behaviors among taxi-driver in Bangkok during COVID-19 pandemic (*R*^2^ = 0.349, *p* = 0.008). The model could predict 34.9% of variance in COVID-19 preventative behavior among taxi drivers.

**Conclusion:**

Taxi drivers should be encouraged to engage in appropriate preventive behaviors against COVID-19, emphasizing the individual and organizational levels. There should be a policy by organizations to promote the implementation of COVID-19 safety control standards to ensure safe working conditions. In addition, appropriate welfare benefits should be provided for taxi drivers, such as loans, personal protective equipment, and access to health services to improve COVID-19 preventive behaviors.

## Introduction

The COVID-19 pandemic is a public health emergency since January 2020 (1). After the COVID-19 outbreak in China, Thailand was the first country that identifies a confirmed case of COVID-19 infection in February 2020 (2). The report on the first case of COVID-19 in Thailand was a taxi driver who worked in the Bangkok area. He reported contact with Chinese tourist passengers in his taxi who had had frequent coughing (2). The first wave of COVID-19 in Thailand began in March 2020; the peak of daily cases in March was under 200; by May 2020, there were 3,042 cumulative cases and 57 deaths (3). In the second wave of COVID-19, there were 21,584 additional cases in 2.5 months (between December 18, 2020, and February 27, 2021) (3).

Public transport systems are a high-risk environment as many peoples are in confined spaces with limited ventilation and no access control to identify infected persons, which may facilitate and accelerate the transmission of COVID-19. The possibility of indirect spread of COVID-19 is not only about disease spread among commuters in close contact but also between drivers and passengers. The World Health Organization (WHO) indicates that taxi drivers and passengers are at high risk of contracting COVID-19 due to frequent contact with passenger service (4). In addition, taxi drivers have been recognized as an occupationally COVID-19 at-risk group (5, 6) due to the nature of their job in a closed space, as they may include passengers who may be affected. For example, taxi drivers in New York have been said to be on the battlefront taking COVID-19 patients to hospitals. As a result, some taxi drivers got ill from occupationally related exposures (7). In Thailand, between May and August 2021, 353 people were infected by public transportation, and 128 were taxi drivers. 36% of public transportation drivers died, and 47% (*N* = 49) of deaths were taxi drivers (8, 9). Persons in this profession are disproportionately negatively impacted by COVID-19. Despite this, taxi drivers can contribute to limiting the spread of COVID-19 (10) by using preventive measures, including cleaning vehicles and surfaces frequently, checking the fever of staff and passengers, improving vehicle ventilation, using masks, and keeping a physical distance (11).

The literature review revealed that most research on taxi drivers focused on socioeconomic and service behaviors. There was no study on behavior in preventing COVID-19 among taxi drivers in Thailand. As a result, this study explored the factors influencing behavior to prevent COVID-19 in taxi drivers.

Rojpaisarnkit's study utilized the PRECEDE–PROCEED model (12) to propose factors influencing disease prevention behaviors. The study found that individual attribute factors, predisposing factors, enabling factors, and reinforcing factors that affect COVID-19 disease prevention behaviors (13). Therefore, in this study, the following factors were selected according to the PRECEDE–PROCEED model (12), which included predisposing factors (i.e., personal characteristics and health beliefs), enabling factors (i.e., accessibility to personal protective equipment for COVID-19, preventive measures against COVID-19), and reinforcing factors (i.e., support in the implementation of COVID-19 preventive behavior). In addition, in this study, health belief was assessed by using the Health Beliefs Model (14) to examine taxi drivers' perception in accordance with COVID-19 preventive behaviors, including perceived susceptibility and severity of COVID-19, perceived of the benefits and barriers to practicing preventive behavior against COVID-19, and motivation to practice preventive behavior.

During the pandemic, the COVID-19 preventive behaviors among taxi drivers in Thailand and the associated factors remain unknown. A greater understanding of factors associated with COVID-19 preventive behaviors among taxi drivers is important to reduce COVID-19 transmission in public transportation in Thailand to enhance the personal safety of both taxi drivers and their passengers during the COVID-19 situation and potentially improve the effectiveness of the public health response. This paper aims to describe factors influencing taxi drivers' COVID-19 preventive behaviors.

The findings of this study may be useful to encourage taxi drivers to implement effective COVID-19 preventive behaviors to prevent the spread of COVID-19 across society and can be used for policy making for public transportation.

## Methods

This cross-sectional survey study was to identify factors associated with COVID-19 preventive behaviors among taxi drivers in Bangkok. This study identified different variables influencing taxi drivers' COVID-19 prevention behaviors by classifying factors into three categories: predisposing, enabling, and reinforcing (12). Personal characteristics and health beliefs, which are internal factors, are considered predisposing factors. External factors that affect the working environment are referred to as enabling and reinforcing factors.

According to the PRECEDE–PROCEED model (12), the predisposing, enabling, and reinforcing constructs in Educational Diagnosis and Evaluation (PRECEDE) behavioral model states that being healthy and having healthy behaviors results from predisposing factors, including knowledge, attitudes, beliefs, values, and perceptions. Enabling factors such as the availability and accessibility of resources or services facilitate the appropriate health behaviors. Last, reinforcing factors help support the desired health behaviors, such as warning, praise, and encouragement (12). This cross-sectional study has been reported using the STROBE guideline (15).

### Participants

Participants in this study were taxi drivers in Bangkok who registered with the Department of Land Transport. They were recruited using systematic random sampling. The sample size calculation was 384 taxi drivers. Anticipating missing data, researchers aimed to recruit 401 participants. At the Department of Land Transport in Bangkok, there was a list of 300 taxi drivers who received the service for vehicle inspection per day, and we want to survey 60 of them. Thus, the sampling interval would be one-fifth. Participants were sampled from every fifth person in the list of 300 customers. To ensure a random sample, researchers used a random start, e.g., a number within the range of the sampling interval. For example, the researcher started with the list's first name and then sampled every fifth person (e.g., 1, 6, 11, …, 300).

Eligible study participants were registered taxi drivers aged 22 years and over. Taxi drivers who have been diagnosed with pulmonary tuberculosis, lung fungal disease, chronic pneumonia, or have to practice anti-infection behaviors regularly due to immunodeficiency or receive immunosuppressive drugs, or are undergoing chemotherapy treatment or those with low white blood cells were excluded from the study. The data was data collected from March to April 2021.

### Measurements

#### Research measurement

Structural questionnaires were developed based on theory and literature reviews for collecting the participants' data for data collection. Three parts of the questionnaire with the Thai version were focused on (1) the predisposing factors, (2) the enabling factors, and (3) the reinforcing factors.

##### Part 1: Predisposing factors

###### Socio-demographic characteristics

Demographic data of the participants were assessed using a 12-item questionnaire consisting of multiple-choice and short-answer questions on the participant's age, gender, marital status, education, work experience, working hours, underlying disease, and treatment of underlying disease, health care coverage, income adequacy, and vehicle ownership.

###### Health beliefs

The 20-item Health Beliefs questions were developed to assess taxi drivers' perceptions. The questionnaire used a Health belief model structure (14) for data collection consisting of six parts, including perceived susceptibility to COVID-19, perceived severity of COVID-19, perceived benefits of practicing preventive behavior against COVID-19, perceived barriers to practicing preventive behavior against COVID-19 and motivation to practicing preventive behavior against COVID-19.

The questions consist of three answer options: agree, fair, and disagree. Scores on each item range from 1 to 3. For each part, the scores of each item were totaled. According to the classification, the mean total scale is calculated by summing all subscale scores and dividing by the number of subscales; total scores on each classification range from 1 to 3. The scores were divided into three categories according to Levin and Rubin's classification (16): low level = 1–1.66, moderate level = 1.67–2.33, and high level = 2.34–3.00. Higher scores reflect a higher perception corresponding to health belief.

##### Part 2: Enabling factors

###### Accessibility to personal protective equipment for COVID-19

The four-item questions were developed to assess taxi drivers' capacity to use personal protection equipment against COVID-19 for themselves and also available for their passengers. The questions used a Likert scale consisting of three answer options: yes, not sure, and no. Scores on each item range from 1 to 3. For each part, the scores of each item were totaled. According to the classification, the mean total scale is calculated by summing all subscale scores and dividing by the number of subscales; total scores on each classification range from 1 to 3. The scores were divided into three categories according to Levin and Rubin's classification (16): low level = 1–1.66, moderate level = 1.67–2.33, and high level = 2.34–3.00. Higher scores reflect a higher capacity to access protection equipment against COVID-19.

###### Preventive measures against COVID-19

The four-item questions were developed to assess the benefit of facilitating COVID-19 preventive behaviors from the Department of Land Transport and other agencies. The questions used a Likert scale consisting of three answer options: Ever, Uncertain, and Never. Scores on each item range from 1 to 3. For each part, the scores of each item were totaled. According to the classification, the mean total scale is calculated by summing all subscale scores and dividing by the number of subscales; total scores on each classification range from 1 to 3. The scores were divided into three categories according to Levin and Rubin's classification (16): low level = 1–1.66, moderate level = 1.67–2.33, and high level = 2.34–3.00. Higher scores reflect a higher benefit of facilitating COVID-19 preventive behaviors from the Department of Land Transport and other agencies.

##### Part 3: Reinforcing factors

###### Support in the implementation of COVID-19 preventive behaviors

The two-item questions were developed to assess the support in implementing COVID-19 preventive behaviors, such as support from the family, co-workers, healthcare workers, and information from the media resource. The questions used a Likert scale consisting of four answer options: never, ever received an extensive, ever received a moderate, and used to get less. Scores on each item range from 0 to 3. For each part, the scores of each item were totaled. According to the classification, the mean total scale is calculated by summing all subscale scores and dividing by the number of subscales; total scores on each classification range from 0 to 3. The scores were divided into three categories according to Levin and Rubin's classification (16): low level = 0–1.0, moderate level = 1.1–2.1, and high level = 2.2–3.00. Higher scores reflect higher support for implementing COVID-19 preventive behaviors.

##### Outcome

###### The COVID-19 preventive behaviors

The 15-item questions were developed to evaluate taxi drivers' use of COVID-19 preventative practices during the last month. The questions consisted of the following topic:

1. Prevention and control of environmental hazards.
2. Eliminating or avoiding hazardous behaviors.
3. Personal hygiene.
4. Personal protection equipment against COVID-19.

The questions used a Likert scale consisting of four answer options: often, sometimes, rarely, and never. Scores on each item range from 0 to 3. For each part, the scores of each item were totaled. According to the classification, the mean total scale is calculated by summing all subscale scores and dividing by the number of subscales; total scores on each classification range from 0 to 3. The scores were divided into three categories according to Levin and Rubin's classification (16): low level = 0–1.0, moderate level = 1.1–2.1, and high level = 2.2–3.00. Higher scores reflect higher COVID-19 preventive behaviors.

Each of the research tools passed a content validity check by three experts in the field of public health. The questionnaire reliability was tested on 40 participants. Parts 1–4 obtained a Content validity index of 0.89, 1.00, 1.00, and 1.00, respectively.

The questionnaire had a total Cronbach's alpha coefficient confidence score of 0.848.

Perceived susceptibility to COVID-19, perceived severity of COVID-19, perceived benefits of practice preventive behavior against COVID-19, perceived barriers of practice preventive behavior against COVID-19, and motivation of practice preventive behavior against COVID-19 had Cronbach's alpha coefficients at confidence levels of 0.880, 0.637, 0.875, 0.783, and 0.779, respectively.

### Data collection

Data were collected between March 29 to April 9, 2021. A 58-items self-administered questionnaire was used to collect data on the socio-demographic characteristics (12 items), health beliefs (20 items), accessibility to personal protective equipment for COVID-19 (4 items), preventive measures against COVID-19 (4 items), support in the implementation of COVID-19 preventive behaviors (2 items), and COVID-19 preventive behaviors (15 items).

### Ethical considerations

This research has been approved by Ethics Review Committee for Human Research at Mahidol University (COA MUPH 2020-157, Dated December 17, 2020). Written informed consent was obtained from all participants. The study was conducted following the Declaration of Helsinki.

### Data analysis

Data were analyzed using SPSS version 18. The data were analyzed descriptively using mean, percentage, standard deviation, maximum, and minimum. The association between predisposing factors, enabling factors, and reinforcing factors with COVID-19 preventive behaviors was analyzed by using analysis of variance and Pearson's Product Moment Correlation. Stepwise multiple regression analysis was used to determine the influencing factors in predicting COVID-19 preventive behaviors of taxi drivers.

## Results

### Predisposing factors

#### Socio-demographic characteristics

Table 1 present the participant characteristics. There were a total of 401 taxi drivers in the study. The majority of participants were male (99.0%), had completed primary school (48.6%), and were married (88.0%). The mean age of participants was 54.2 ± 8.96 years. The average daily income was 451 ± 199.9 baht, and 52.1% had inadequate income. 93.8% of participants worked full-time and 77.1% of participants drove taxis for more than 8 hrs per day. The average year of taxi drivers' experience was 15.1 ± 9.06 years. Over 80% of participants had private ownership of taxis. In total, 69.3% were healthy, while the remaining 31.7% had at least one chronic disease, such as diabetes and hypertension; most were treated (97.8%). The Universal Health Coverage Plan was used by 61.8% of participants for health insurance. Tables 2–4 presents the descriptive statistics for the predisposing factors, enabling factors, and reinforcing factors among taxi drivers.

**Table 1:** Participant characteristics (*N* = 401).

	***N***	**%**	**Mean**	**SD**	**Range**
**Gender (*****n*** = **401)**					
Male	397	99.0			
Female	4	1.0			
**Age (years) (*****n*** = **401)**			54.2	8.9	23–83
< 31	4	1.0			
31–40	27	6.7			
41–50	89	22.2			
51–60	190	47.4			
61–70	79	19.7			
**Marital status** **(*****n*** = **401)**					
Single	23	5.7			
Married	353	88.0			
Widow	4	1.0			
Divorce	14	3.5			
Separate	7	1.7			
**Education level**					
Primary school	195	48.6			
Secondary school	159	39.7			
Vocational certificate	30	7.5			
Bachelor's degree and above	17	4.2			
**Work experience**					
**Taxi driver as a** **full-time job** **(year) (*****n*** = **377)**			15.1	9.06	0–58
< 10	110	29.17			
10–20	187	49.61			
>20	80	21.22			
**Taxi driver as a** **part-time job** **(year) (*****n*** = **50)**			11.2	8.6	0–31
< 10	24	48.0			
10–20	19	38.0			
>20	7	14.0			
**Working hours** **(hours/day)** **(*****n*** = **401)**			11	3	3–24
≤ 8	92	22.9			
>8	309	77.1			
**Underlying disease** **(*****n*** = **401)**					
No	278	69.3			
Yes	93	23.2			
Unknown	30	7.5			
**Treatment of** **underlying disease** **(*****n*** = **93)**					
					
No	2	2.15			
Yes	91	97.8			
**Health care** **coverage** **(*****n*** = **401)**					
Universal health coverage	248	61.8			
Self-paid	83	20.7			
Social security scheme	49	12.2			
Civil service medical benefit scheme	17	4.2			
Others	4	1.0			
**Income (baht/day)** **(*****n*** = **401)**			451	199.9	100–1,500
≤ 300	158	39.4			
300–1,000	242	60.3			
≥1,000	1	0.2			
**Income adequacy** **(*****n*** = **401)**					
Adequate	192	47.9			
Not adequate	209	52.1			
**Owner of taxi** **(*****n*** = **401)**					
Self-owner	328	81.8			
Regular rental	70	17.5			
Non-regular rental	3	0.7			
**Access to** **information (*****n*** = **401)**					
Social media	180	44.89			
Radio	131	32.67			
Television	86	21.45			
Others	4	1.0			

**Table 2:** Descriptive statistics of predisposing factors among taxi drivers (*N* = 401).

**Predisposing factors**	**Low**		**Moderate**		**High**	
	**Number**	**%**	**Number**	**%**	**Number**	**%**
Overall health belief	1	0.2	5	1.2	395	98.5
$\bar{x}$ = 2.8, SD = 0.193, min = 1, max = 3						
Perceived susceptibility to COVID-19	9	2.2	15	3.7	377	94.1
$\bar{x}$ = 2.89, SD = 0.316, min = 1, max = 3						
Perceived severity of COVID-19	2	0.5	6	1.5	393	98.0
$\bar{x}$ = 2.92, SD = 0.231, min = 1, max = 3						
Perceived benefits of practicing preventive behavior against COVID-19	2	0.5	6	1.5	393	98.0
$\bar{x}$ = 2.94, SD = 0.203, min = 1, max = 3						
Perceived barriers to practicing preventive behavior against COVID-19	29	7.2	70	17.5	302	75.3
$\bar{x}$ = 2.65, SD = 0.528, min = 1, max = 3						
Motivation to practice preventive behavior against COVID-19	1	0.2	2	0.5	398	99.3
$\bar{x}$ = 2.98, SD = 0.139, min = 1, max = 3						

**Table 3:** Descriptive statistics of enabling factors among taxi drivers (*N* = 401).

**Enabling factors**	**Low**		**Moderate**		**High**	
	**Number**	**%**	**Number**	**%**	**Number**	**%**
Accessibility to personal protective equipment for COVID-19	1	0.2	27	6.7	373	93.0
$\bar{x}$ = 2.89, SD = 0.281, min = 1.5, max = 3						
Overall preventive measures against COVID-19	315	78.6	74	18.5	12	3.0
$\bar{x}$ = 1.44, SD = 0.381, min = 1, max = 3						
Preventive measures against COVID-19 from the department of land transport	373	93.0	15	3.7	13	3.2
$\bar{x}$ = 1.20, SD = 0.395, min = 1, max = 3						
Preventive measures against COVID−19 from other agencies	277	69.1	69	17.2	55	13.7
$\bar{x}$ = 1.67, SD = 0.515, min = 1, max = 3						

**Table 4:** Descriptive statistics of reinforcing factors among taxi drivers (*N* = 401).

**Reinforcing factors**	**Low**		**Moderate**		**High**	
	**Number**	**%**	**Number**	**%**	**Number**	**%**
Support from family	20	5.0	103	25.7	278	69.3
$\bar{x}$ = 2.56, SD = 0.656, min = 0, max = 3						
Support from co-workers	87	21.7	174	43.4	140	34.9
$\bar{x}$ = 1.97, SD = 0.859, min = 0, max = 3						
Support from healthcare workers.	330	82.3	39	9.7	32	8.0
$\bar{x}$ = 0.601, SD = 0.899, min = 0, max = 3						

#### COVID-19 health belief

Regarding COVID-19 health beliefs, high perceived susceptibility and perceived severity of COVID-19 were reported by 94.1% and 98.0% of participants, respectively. In addition, 98.0% of participants had a high perception of the benefits of practicing preventive behavior against COVID-19, 75.3% of the participant had a high perception of the barriers to practicing preventive behavior against COVID-19, and 99.3% of participants had high motivation to practice preventive behavior against COVID-19.

### The enabling factors

#### Accessibility to personal protective equipment for COVID-19 and preventive measures against COVID-19

It was found that participants had a high level of accessibility to personal protective equipment for COVID-19 (93.0%). However, COVID-19 prevention measures that help facilitate COVID-19 prevention behaviors were at a low level (78.6%). In addition, the preventive measures against COVID-19 from the Department of Land Transportation were at a low level (93.0%), and preventive measures against COVID-19 from other agencies were at a low level (69.1%).

### The reinforcing factors

#### Support in the implementation of COVID-19 preventive behaviors

The majority of the participants received a high level of family support (69.3%), moderate levels of co-worker support (43.4%), and low levels of healthcare worker support (82.3%). In addition, 44.9% of participants received information about COVID-19 *via* social media channels.

#### COVID-19 preventive behaviors

Table 5 presents descriptive statistics of COVID-19 preventives behaviors among taxi drivers. Most participants had a high level of overall COVID-19 preventive behaviors (74.3%). When each item was considered, most of them had a high level of prevention (83.3%) and control of environmental hazards and personal hygiene (73.6%). Eliminating or avoiding hazardous behaviors (54.1%) and using personal protection equipment against COVID-19 (68.1%) were at a moderate level.

**Table 5:** Descriptive statistics of COVID-19 preventives behaviors among taxi drivers (*N* = 401).

**COVID-19 preventive behaviors**	**Low**		**Moderate**		**High**	
	**Number**	**%**	**Number**	**%**	**Number**	**%**
Overall COVID-19 preventive behaviors	0	0	103	25.7	298	74.3
$\bar{x}$ = 2.38, SD = 0.95, min = 1.2, max = 3						
Prevention and control of environmental hazards	3	0.7	64	16.0	334	83.3
$\bar{\bar{x}\;}$ = 2.64, SD = 0.465, min = 1, max = 3						
Eliminating or avoiding hazardous behaviors	61	15.2	217	54.1	123	30.7
$\bar{x}$ = 1.99, SD = 0.75, min = 0, max = 3						
Personal hygiene	13	3.2	93	23.2	295	73.6
$\bar{x}$ = 2.47, SD = 0.536, min = 0.667, max = 3						
Personal protection equipment against COVID-19	1	0.2	273	68.1	127	31.7
$\bar{x}$ = 2.19, SD = 0.474, min = 0.667, max = 3						

### Factors associated with COVID-19 preventive behaviors

#### Predisposing factors

Marital status, underlying disease, and income adequacy were statistically significantly associated with COVID-19 preventive behaviors (*F* = 6.666, 7.536, 26.063, *p* < 0.01, respectively). Age, education, work experiences, working hours, and vehicle ownership were not associated with COVID-19 preventive behaviors. Overall health belief scores were positively associated with COVID-19 preventive behaviors (*r* = 0.474, *p* < 0.01). When each aspect was considered, it was found that perceived susceptibility to COVID-19, perceived severity of COVID-19, perceived benefits of practicing preventive behavior against COVID-19, perceived barriers to practicing preventive behavior against COVID-19 and motivation to practicing preventive behavior against COVID-19 had a significant positive association with COVID-19 preventive behaviors (*r* = 0.234, 0.372, 0.373, 0.351 and 0.262, *p* < 0.01, respectively), as seen in Table 6.

**Table 6:** Correlations coefficient among predisposing factors, enabling factors, and reinforcing factors and COVID-19 preventive behaviors among taxi drivers in Bangkok (*N* = 401).

**Predisposing factors**	***F***	**Sig**
Marital status	6.666^*^	< 0.01
Education level	0.840	0.473
Underlying disease	7.536^*^	< 0.01
Sufficiency of income	26.063^*^	< 0.01
Car owner	1.193	0.304
**Predisposing factors**	***r***	**Sig**
Age	−0.021	0.678
Working experiences	0.017	0.739
Working hours	0.071	0.155
Overall health belief	0.474^*^	< 0.01
Perceived susceptibility to COVID-19	0.234^*^	< 0.01
Perceived severity of COVID-19	0.372^*^	< 0.01
Perceived benefits of practicing preventive behavior against COVID-19	0.373^*^	< 0.01
Perceived barriers to practicing preventive behavior against COVID-19	0.351^*^	< 0.01
Motivation to practice preventive behavior against COVID-19	0.262^*^	< 0.01
**Enabling factors**	***r***	**Sig**
Accessibility to personal protective equipment for COVID-19	0.339^*^	< 0.01
Overall preventive measures against COVID-19	0.168^*^	< 0.01
Preventive measures against COVID-19 from the department of land transport	0.052	0.258
Preventive measures against COVID-19 from other agencies	0.209^*^	< 0.01
**Reinforcing factors**	***r***	**Sig**
Support from family	0.165	< 0.01
Support from co-workers	0.298	< 0.01
Support from healthcare workers	0.141	< 0.01

* p < 0.01.

#### Enabling factors

The accessibility to personal protective equipment for COVID-19 and preventive measures against COVID-19 had a statistically significant positive association with COVID-19 preventive behaviors (*r* = 0.339, 0.168; *p* < 0.01, respectively). The preventive measures against COVID-19 from other agencies, such as Bangkok Metropolitan, and the Ministry of Public Health, had a positive correlation with COVID-19 preventive behaviors (*r* = 0.209, *p* < 0.01). Interestingly, no association was found between the preventive measures against COVID-19 from Department of Land Transport and COVID-19 preventive behaviors, as seen in Table 6.

#### Reinforcing factors

Support for the practice of COVID-19 preventive behaviors from family, co-workers, and healthcare workers was found to have a statistically significant positive relationship with COVID-19 preventive behaviors (*r* = 0.165, 0.298, and 0.141, *p* < 0.01, respectively). However, the information received about COVID-19 did not correlate with COVID-19 preventive behaviors, as seen in Table 6.

A stepwise multiple regression analysis was used to predict taxi drivers' COVID-19 preventive behaviors. Seven factors were used to predict taxi drivers' COVID-19 prevention behaviors, including; perceived benefits of practicing preventive behavior against COVID-19, perceived barriers to practicing preventive behavior against COVID-19, accessibility to personal protective equipment for COVID-19, support from co-workers, perceived severity of COVID-19, income adequacy, and preventive measures against COVID-19 from other agencies (beta = 0.183, 0.164, 0.185, 0.120, 0.188, −0.130, 0.110, respectively), as seen in Table 7. The multiple linear regression analysis explained 34.9% of the COVID-19 preventive behaviors among taxi drivers. The multiple regression equation was COVID-19 preventive behaviors (Y) = −11.694 + 1.337 (perceived benefits of practicing preventive behavior against COVID-19) + 0.459 (perceived barriers to practicing preventive behavior against COVID-19) + 0.976 (accessibility to personal protective equipment for COVID-19 + 0.413 (support from co-workers) + 1.207 (perceived severity of COVID-19) −1.538 (income adequacy) + 0.316 (preventive measures against COVID-19 from other agencies).

**Table 7:** Coefficient multiple regression of factors in predicting the preventive behavior of COVID-19 among taxi drivers in Bangkok.

**Variables**	**B**	**Std.** **error**	**Beta**	**p-value**	***R* ^2^**	***R*^2^ change**
Perceived benefits of practicing preventive behavior against COVID-19	1.337	0.339	0.183	< 0.001	0.139	0.139
Perceived barriers to practicing preventive behavior against COVID-19	0.459	0.124	0.164	< 0.001	0.216	0.077
Accessibility to personal protective equipment for COVID-19	0.976	0.226	0.185	< 0.001	0.270	0.054
Support from co-workers	0.413	0.153	0.120	0.007	0.298	0.028
Perceived severity of COVID-19	1.207	0.299	0.188	< 0.001	0.322	0.025
Income adequacy	−1.538	0.504	−0.130	0.002	0.339	0.016
Preventive measures against COVID-19 from other agencies	0.316	0.123	0.110	0.011	0.349	0.011
Constant = −11.694; *F* = 30.152; *R*^2^ = 0.349; *p* = 0.008						

## Discussion

This cross-sectional study found that most taxi drivers in Bangkok had COVID-19 preventive behaviors at a high level (74.3%) which is consistent with previous studies on preventing respiratory problems in other occupations in Thailand (17–22). In addition, the taxi driver in Thailand followed the safety practice for COVID-19 prevention, such as mask-wearing and frequent hand washing with soap or hand sanitizer. In contrast, in a study in Ethiopia, public taxi drivers had poor COVID-19 prevention practices and were highly dependent on traditional medicines and religious practices (23).

We found that marital status, underlying disease, and income adequacy were associated with COVID-19 preventive behaviors. The marriage status of taxi drivers was associated with COVID-19 preventive behaviors. Taxi drivers who were married had a greater predisposition to limiting the transmission of COVID-19 than those who were single/ widowed/divorced and separated. If a taxi driver gets COVID-19 infection, he may spread the virus to other family members. Moreover, COVID-19 may affect households in many ways, such as loss of income. Additionally, married persons were more likely to have a caregiver and an interest in marriage health care, which was supported by Pender's assumption that marital health care is a significant source of health promotion (24).

The underlying disease was associated with COVID-19 preventive behaviors, which may be related to the fact that COVID-19 frequently caused severe symptoms in people with underlying diseases such as diabetes, hypertension, obesity, and heart disease. In this study, 23.2% of taxi drivers had a chronic illness. Thus, taxi drivers with underlying diseases showed more protective behaviors against COVID-19 than taxi drivers who did not have an underlying disease. In addition, Thailand had proactive measures to monitor and prevent high-risk groups, especially people with an underlying disease, by providing knowledge and increasing awareness of COVID-19 infection through various media. Therefore, taxi drivers also create new normal behaviors to prevent COVID-19 infection.

Income was associated with COVID-19 preventive behaviors. Consistent with a previous study among older adults in urban communities in Thailand, adequate income was associated with good COVID-19 preventive behaviors (25). In contrast, Wang and Tang's study reported that adequate income is associated with health behaviors (26). However, some taxi drivers have faced financial difficulty during the COVID-19 pandemic (27). Without sufficient support for personal protective equipment against COVID-19 (masks and hand sanitizers) from other authorities, taxi drivers with inadequate incomes may not be able to access personal protective equipment against COVID-19.

Interestingly, education level was not associated with COVID-19 preventive behaviors, consistent with the previous study among older adults in urban communities in Thailand (25). Previous research has revealed that socioeconomic status indicators, such as education levels, were associated with health behaviors (28). For example, highly educated people can take care of themselves, protect themselves against risk factors (29) and seek health information to manage their chronic illnesses (30). This result contrasts with a study among Chinese residents in Hubei, where knowledge was significantly associated with attitudes and preventive practices toward COVID-19 (31). Kebede et al.'s study revealed that knowledge could predict hand washing and avoiding handshaking (32). COVID-19 is the disease caused by the new coronavirus that has just emerged globally, and everyone has to pay attention to their health and infection prevention. As a result, preventing the spread of COVID-19 requires everyone to adapt to a new normal, and there were strict disease control and prevention measures in cooperation with the government. In addition, taxi drivers' agencies are responsible for monitoring and guiding how to prevent COVID-19 infections for professional taxi drivers.

Regarding health beliefs and COVID-19 preventive behaviors, perceived susceptibility, severity, benefits, barriers, and health motivation were associated with COVID-19 preventive behaviors. Taxi drivers were at a high risk of COVID-19 infection compared to other populations. Continuous media reports about COVID-19's severity and impact on taxi drivers, such as the transmission of infection to family members, loss of income, or death, make taxi drivers aware of the disease's severity. Thus, they practice COVID-19 preventive behaviors.

We found that the accessibility to personal protective equipment for COVID-19 and preventive measures against COVID-19 implemented by the Department of Land Transport and other agencies were associated with the prevention of COVID-19 behavior among taxi drivers in Bangkok. In addition, a previous study among older adults in urban communities in Bangkok also found that access to protective material was associated with COVID-19 preventive behaviors (25).

We found that support from family, co-workers, and healthcare providers were associated with COVID-19 preventive behaviors among taxi drivers. Interestingly, no association was found between receiving information about COVID-19 from the media and taxi drivers' COVID-19 preventive behaviors. As Thailand was the first country outside China to report a confirmed case of COVID-19, Thai society was interested in reading any news reported by all types of media (33). However, the dissemination of misleading information led to adverse effects, such as rumors, false knowledge, fear, disguised history of exposure to risk groups of COVID-19, and social stigma (33). These adverse effects caused panic in society rather than raising awareness of practices to prevent further infection in the community (34).

The findings of the study revealed that multiple factors could predict COVID-19 preventive behaviors. The combination of predisposing, enabling, and reinforcing factors could predict COVID-19 preventive behaviors among taxi drivers in Bangkok. This finding was statistically significant (*p* < 0.001), with 34.9 percent (R^2^ = 0.349). The predisposing factors that significantly predict COVID-19 preventive behaviors are perceived COVID-19 severity, perceived benefits of practicing preventive behavior against COVID-19, perceived barriers to practicing preventive behavior against COVID-19, and income adequacy. The enabling factors were accessibility to personal protective equipment for COVID-19 and preventative measures against COVID-19 from other agencies. The reinforcing factor was co-worker support.

The findings of this study corroborated the conceptual framework, PRECEDE^−^PROCEED Model, which states that health behavior is influenced by internal and external factors (12). Therefore, taxi drivers should be encouraged to engage in appropriate preventive behaviors against COVID-19, with an emphasis on the individual, by raising awareness of the perceived severity of COVID-19, the perceived benefits of practicing preventive behavior against COVID-19, and the perceived barriers to practicing preventive behavior against COVID-19, including having income adequacy. Additionally, external factors should be encouraged by supporting taxi drivers in practicing COVID-19 preventive behaviors by supporting taxi drivers in receiving personal prevention equipment against COVID-19. Training on how to prevent the spread of COVID-19 includes an annual physical examination, influenza vaccine, chest x-ray, publicity about the posting of warning signs for taxi drivers to prevent the spread of COVID-19, and support from others, particularly co-workers, which helps promote consistent behaviors for health and productive work life.

The findings of this study suggested that there should be a policy by organizations in the responsibility of taxi driver supervision to promote the implementation of COVID-19 safety control standards to ensure safe working conditions. Protective equipment such as masks, hand wash gel, and a car barrier should be provided. Taxi associations and taxi garages should regularly increase awareness of COVID-19 prevention, such as by communicating the benefits of COVID-19 prevention and the severity of COVID-19. Friends remind friends activities should also be developed to increase support from co-workers. Safe taxis should be promoted through the taxi service business by having loans and protective equipment as welfare benefits. The Occupational Health Nurses Association should be involved in developing taxi driver health policies and healthcare services to improve COVID-19 preventive behaviors.

Further study in COVID-19 preventive behaviors among other public transportation drivers, such as vans and buses, should be conducted to identify factors influencing preventive behaviors in COVID-19 and other respiratory diseases. In addition, qualitative research to obtain insights into different aspects and to develop policy proposals should be conducted to provide suitable policy suggestions relevant to the issue and career context.

## Conclusion

The present findings revealed that income adequacy, good support from family, co-workers, and healthcare professionals, perceived susceptibility, severity, benefits, barriers, and health motivation, and accessibility to personal protective equipment for COVID-19 and preventative measures against COVID-19 from other agencies were associated with COVID-19 preventive behaviors among taxi-driver in Bangkok during COVID-19 pandemic. Therefore, taxi drivers with COVID-19 preventive behaviors will have a low risk of getting COVID-19 infection. Based on these results, healthcare providers should consider health beliefs, social support, and access to personal protective equipment when developing interventions to improve COVID-19 preventive behaviors. Also, appropriate measures or policies should be developed to maintain physical distancing in the public transport system, and necessary information or training should be provided to the staff and passengers.

## Data availability statement

The raw data supporting the conclusions of this article will be made available by the authors, without undue reservation.

## Ethics statement

The studies involving human participants were reviewed and approved by Ethics Review Committee for Human Research at Mahidol University (COA MUPH 2020-157, Dated December 17, 2020). The patients/participants provided their written informed consent to participate in this study.

## Author contributions

AD, WK, SK, and JS contributed to the design and implementation of the research and to the analysis of the results. AD and WK wrote the manuscript with input from all authors. All authors contributed to the article and approved the submitted version.
